# Research on crack evolution law and macroscopic failure mode of joint Phyllite under uniaxial compression

**DOI:** 10.1038/s41598-021-83571-9

**Published:** 2021-02-18

**Authors:** Jiangbo Xu, Dongyang Fei, Yanglin Yu, Yilun Cui, Changgen Yan, Han Bao, Hengxing Lan

**Affiliations:** 1 School of Highway, Chang’an University, Xi’an, 710064 Shaanxi China; 2 Gansu Province Water Conservancy and Hydropower Survey and Design Institute Co. LTD, Lanzhou, 730000 Gansu China; 3 Institute of Geographic Sciences and Natural Resources Research, CAS, Beijing, 100101 China

**Keywords:** Natural hazards, Solid Earth sciences

## Abstract

In order to explore the fracture mechanism of jointed Phyllite, the TAJW-2000 rock mechanics test system is used to carry out uniaxial compression tests on different joint inclination Phyllites. The influence of joint inclination of Phyllite failure mode is discussed, and the progressive failure process of Phyllite is studied. The test results show that the uniaxial compressive strength anisotropy of jointed Phyllite is remarkable. As the inclination increases, it exhibits a U-shaped change; When 30° ≤ α ≤ 75°, the tensile and shear failures along the joint inclination mainly occurs. the joint inclination controls the failure surface form of the Phyllite; The crack initial stress level of the joint Phyllite is 0.30–0.59σf, the crack failure stress level is 0.44–0.86σf. When α = 90°, the σ_cd_ value is the largest, and σ_cd_ with α = 90° can be used as the maximum reliable value of uniaxial compressive strength of Phyllite. Using the theory of fracture mechanics, it is analyzed that under uniaxial compression of the rock, the crack does not break along the original crack direction, but extends along the direction at a certain angle to the original crack. The joint effect coefficient is proposed to show the influence of the joint inclination on the uniaxial compressive strength of the phyllite. Both the test and simulation results show that when the joint inclination is 60°, the joint effect coefficient is the largest. The compressive strength is the smallest. Numerical simulation analyses the crack evolution law of phyllite under different joint inclination under uniaxial compression, which verifies that there are different failure modes of joint phyllite under uniaxial compression.

## Introduction

As a kind of heterogeneous and anisotropic material, Phyllite has a large number of joints inside due to long-term geological conditions. The integrity of the rock mass is poor, and the strength and failure characteristics of the Phyllite in different joint faces are significantly different, the anisotropy of the Phyllite is more prominent. The research on the rupture mechanism of the Phyllite in the joint plane and the gradual destruction process of the Phyllite in different joints are of great significance to ensure the stability of the Phyllite rock engineering.

The anisotropy study of the rupture mechanism of Phyllite has attracted wide attention at home and abroad. Bieniawski^1^ established a mechanism of brittle fracture of rock in compression and tension, propounded hypothesis on the mechanism of rock fracture and presented the results of fracture studies on specimens of different shapes and subjected to different loading conditions. Taliercio and Landriani^2^ established a model which enables the description with fair accuracy of the ultimate behaviour of layered rocks submitted to triaxial tests, varying both the orientation of the principal stresses to the layers and the confining pressure. Ramamurthy et al.^3^ conducted a compression test on quartz Phyllite, carbonaceous Phyllite and mica Phyllite. The phyllite physics characteristic curve of the confining pressure and the locating angle function is obtained, and the degree of anisotropy is quantified according to the measured parameters. Eberhardt^4^ found grain size had only a minor effect on the stress at which new cracks initiated and crack initiation thresholds was more dependent on the strength of the constituent minerals. Nicksiar and Martin^5^ found that the heterogeneity introduced by the grain size distribution had the most significant effect on peak strength and crack initiation stress. Tien and Kuo^6^ proposed a new failure criterion for the transversely isotropic rocks, the predicted strength behaviors of the transversely isotropic rocks agree well with the experimental data from various investigators and the accuracy and applicability of the proposed empirical failure criterion are demonstrated. Zheng et al.^7^ revealed the relationship between the micro-fracture form, fracture mechanism and mineral composition of phyllite rocks and pointed out that the sericite phyllite rock is mainly composed of microscopic fractures along the crystal face, which is a typical microscopic brittle shear. The siliceous platy phyllite rock has both the fracture and the fracture along the crystal fracture, and the shear micro-fracture form of the crystal-cutting, which belongs to the micro-fracture mechanism of the pull and shear fracture and pointed out that the anisotropy of phyllite is more sensitive to uniaxial compressive strength. Wang et al.^8^ carried out a triaxial compression test and analyzed the whole process of Phyllite destruction. It was found that the failure modes of Phyllite in the range of 0° to 50° are divided into tension-shear composite failure, cross-section structural plane shear and composite failure along joint surface, shear sliding failure along the joint surface, and as the confining pressure increases, the anisotropy of the Phyllite decreases. Wu et al.^9–12^ compared the strength and deformation characteristics of four typical Phyllites in the northern section of Longmenshan tunnel under different loading azimuths, and found that the anisotropy rate of sericite Phyllite is the largest, reaching 0.48. The failure mode of Phyllite can be divided into four types: layered surface slip shear failure, compression and shear failure, tensile failure and composite failure. In addition, damage expansion is a typical failure mode of Phyllite, and Phyllite generally extends before the peak. Zhou et al.^13^ studied the X-ray diffraction, thin-film identification, uniaxial and triaxial compression tests with schist fracture Phyllite as the research object. When the angle of the structural plane changes from 0° to 90°, the elastic modulus, compressive strength, cohesion and internal friction angle of the slab Phyllite decrease first and then increase, showing a V-shaped distribution law. For the study of progressive failure of rock, Martin^14^, Zhang^15,16^, Li et al.^17^ believe that the compression failure process of brittle rock can be divided into fracture compaction stage, elastic stage, crack stability expansion stage, crack acceleration expansion stage and post-peak stage. The peak intensity σf is related to the experimental boundary conditions such as the sample size, and σ_cd_ is the reliable peak intensity of the rock.

Behnia et al.^18,19^ presents boundary element method based on the displacement discontinuity formulation to solve general problems of interaction between hydraulic fracturing and discontinuities. Besides, They compared the two equivalent strength methods of “weighted average method” and “intensity volume ratio” for the problem of equivalent strength of rock composed of soft rock layer and strong rock layer, and the results showed that the equivalent strength obtained by “intensity volume ratio” was more appropriate. Aboutaleb et al.^20^ use the non-destructive method to propose multiple linear regression equations between the destructive parameters and the non-destructive parameters, and predicts the uniaxial compression strength of the rock through the conventional parameters of the rock. Mokhtari et al.^21^ used three artificial intelligence technologies, local linear neural fuzzy (LLNF), artificial neural network (ANN) and hybrid optimization algorithm of rhododendron—Artificial neural Network (COA-ANN) to estimate the uniaxial compression strength and the static Young's modulus of limestone. Evaluating the correlation coefficients and error criteria resulting from the three methods used demonstrates the superiority of LLNF method to ANN and COA-ANN methods. Alneasan et al.^22^ has studied in detail the existence of inclined internal interface cracks between two different rock layers under compression and tension. The results show that the interface crack propagation may occur in the harder or weaker layer, depending on the comparison of elastic stiffness and crack inclination. Mahmoud et al.^23^ predicts the path of crack propagation by defining three stress states, namely uniaxial tensile stress, uniaxial compressive stress and biaxial compressive stress. Compared with the experimental results, it is found that as the crack inclination angle increases, the length of the crack propagation path increases, while the slope of the crack propagation path decreases. Marji et al.^24,25^ using cubic elements formulation to solve crack problems in two dimensional displacement discontinuity method, which more efficiently improve the accuracy of the conventional displacement discontinuity method, and uses the modified displacement discontinuity method to analyze the mechanism of quasi-static crack branching in brittle solids, and verifies the accuracy and applicability of the method. Nikadat et al.^26^ study the stress distribution around the tunnels excavated in jointed rock masses by TFSDDM, The numerical analysis revealed that both the dip angle and spacing of joints have important influences on stress distribution on tunnel walls.

The existing references have focused on the study of the strength, deformation and failure modes of Phyllites with joint angles of 0°, 45° and 90°. The angle of the joint surface changes greatly, and the angle of the joint surface cannot be refined. In addition, there is a lack of research related to the progressive destruction of Phyllite. Most jointed rocks are studied mainly through laboratory experiments, and the research methods are too simple. Therefore, in this paper, uniaxial compression tests are carried out on Phyllites with joint angles of 0°, 15°, 30°, 45°, 60°, 75°, and 90°, investigating the anisotropy of its failure mode and study of the asymptotic failure process of jointed Phyllit systematically. The rock failure mechanism was analyzed from the perspective of fracture mechanics. RFPA was used to simulate the strength of Phyllite under uniaxial compression to explore the influence of joint inclination Angle on the strength of phyllite. Through mutual verification of laboratory test and numerical simulation, the variation law of strength of phyllite under uniaxial compression was fully explained.

## Sample preparation and test equipment

In order to study the deformation and failure characteristics and macroscopic failure modes of jointed phyllite under uniaxial compression, the author excavated phyllite from Jiujiang City, Jiangxi Province, China, and processed it into standard specimens at different inclination angles, and carried out phyllite uniaxial compression tests. Obtain the mechanical properties of phyllite.

### Material condition

**Figure 1:**
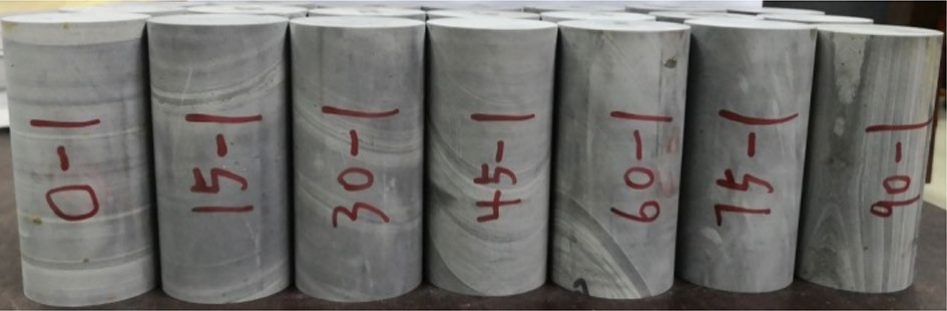
Phyllite samples.

The sample is the Phyllite taken from the drilling, and is made into a cylindrical test piece according to the test standard, with a diameter of 50 mm and a length of 100 mm. According to the relationship between the joint surface and the horizontal plane, the dip angle α of the joint surface of the Phyllite sample is 0°, 15°, 30°, 45°, 60°, 75°, 90°(As shown in Fig. 1).

### Test plan

**Figure 2:**
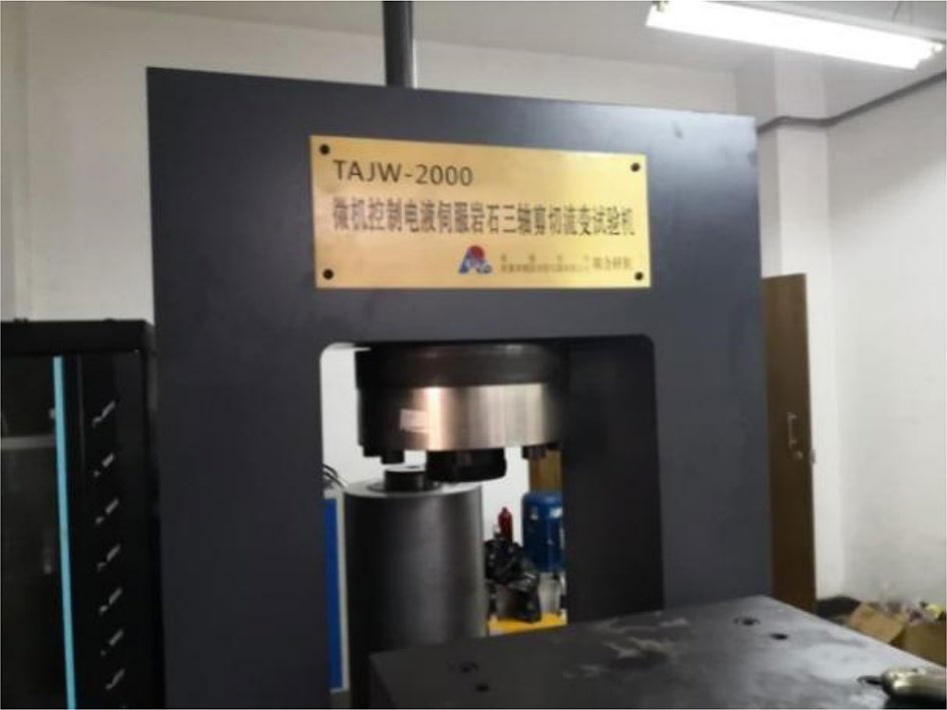
TAJW-2000 triaxial shear rheometer.

**Figure 3:**
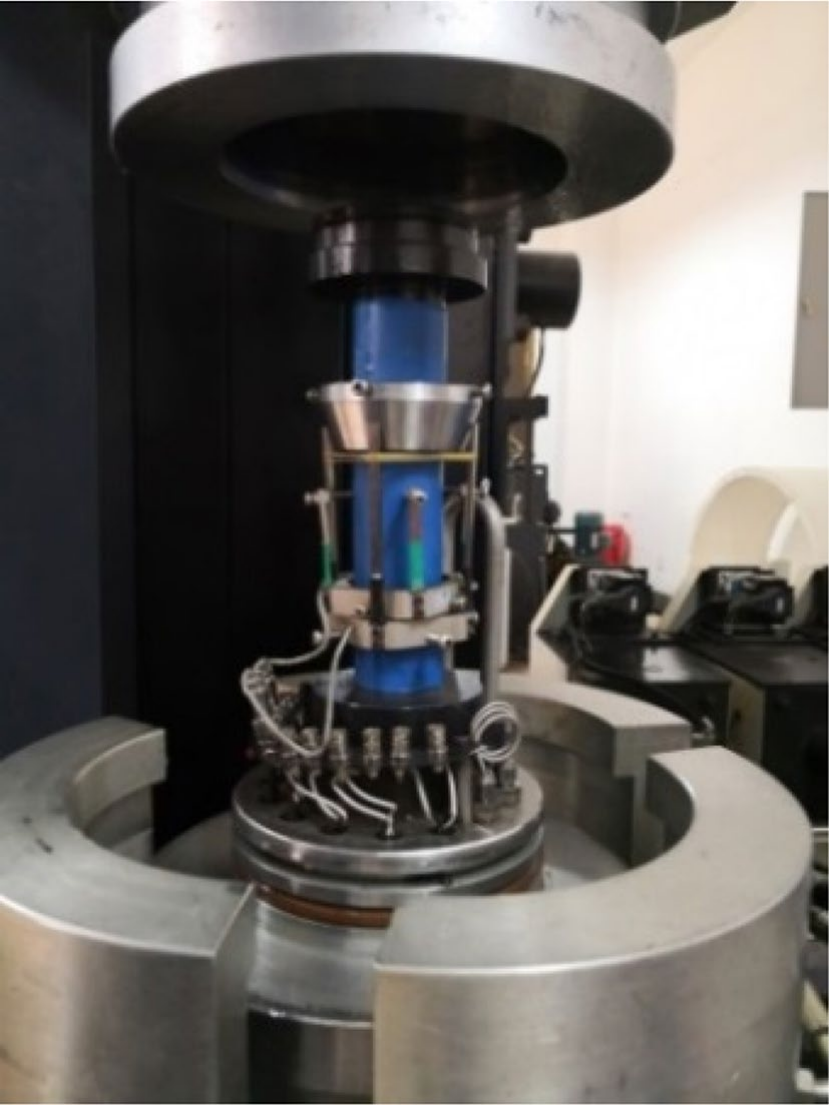
Sample installation diagram.

The test was carried out on a microcomputer controlled servo rock triaxial shear rheometer (TAJW-2000), As shown in Fig. 2. The radial and radial strains of the loading process were measured by radial and circumferential extensometers, and the whole process of rock failure was tracked to obtain the full stress–strain curve of the rock, As shown in Fig. 3. In order to minimize the effect of the end effect on the test results, Vaseline is applied between the upper and lower ends of the sample and the compact when installing the sample, and then fixed with self-adhesive tape. The uniaxial test uses a displacement control method in which the appropriate speed is first applied to bring the sample into contact with the instrument loading shaft, and then the loading rate is controlled to 0.1 mm/min until the sample is broken.

## Test results and analysis

According to the test results, the stress–strain curves of different joint phyllites under uniaxial compression are compared and analyzed, the stress change law of joints on phyllite is analyzed, and the macroscopic failure modes of different joint phyllites under uniaxial compression are discussed., Studied the fracture development stage of joint phyllite under uniaxial compression.

### Analysis of stress–strain process of Phyllite

**Figure 4:**
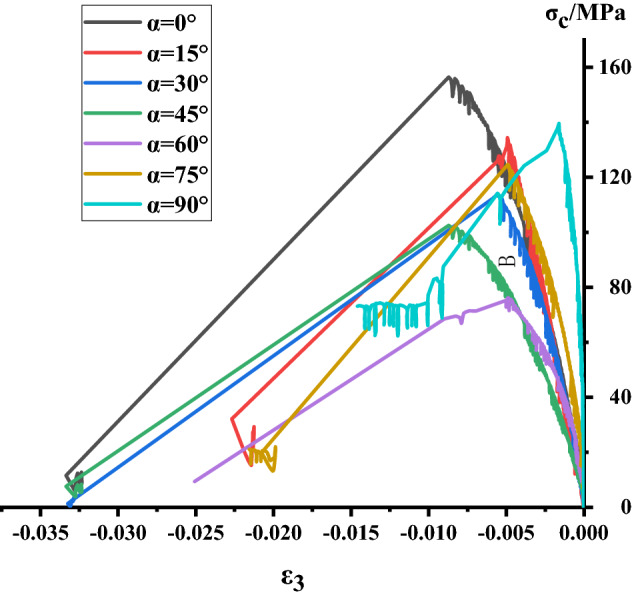
σ_c_–ε_3_ curve of Phyllite.

**Figure 5:**
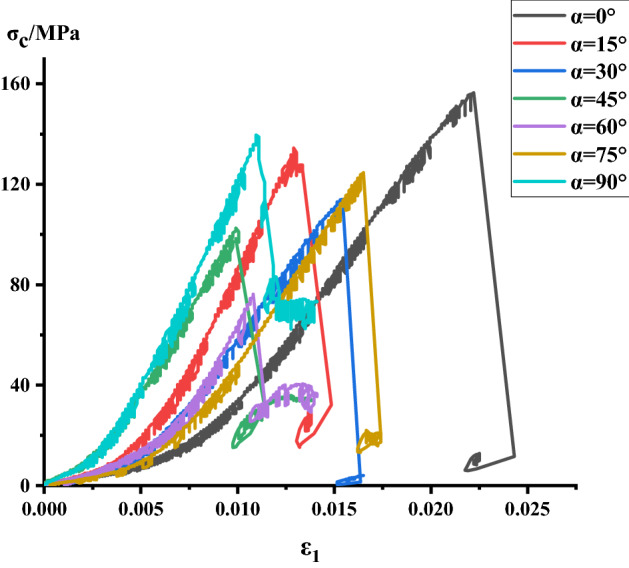
σ_c_–ε_1_ curve of Phyllite.

**Figure 6:**
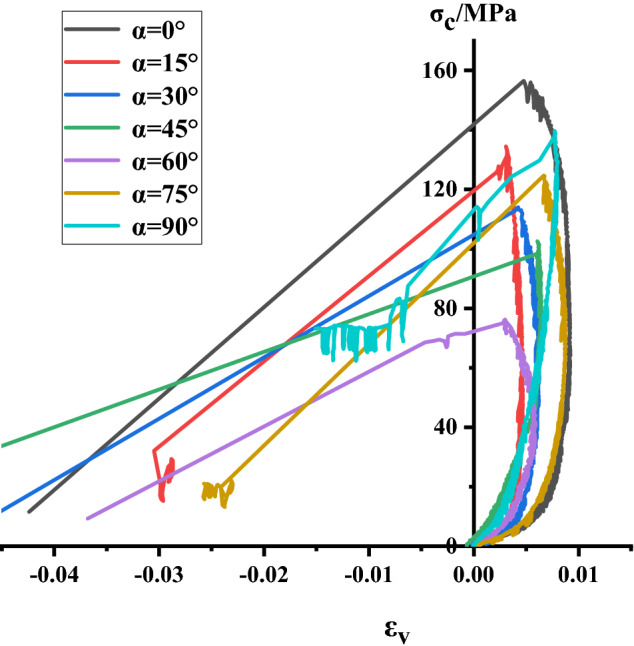
σ_c_–ε_v_ curve of Phyllite.

According to the above test scheme, the classical stress–strain curves of uniaxial compression of Phyllite under different joint angles were successfully obtained. and compared σ_c_-ε_1_, σ_c_-ε_3_, σ_c_-ε_v_ of different joint plane dip Phyllites, as shown in Figs. 4, 5 and 6. The volumetric strain is calculated as $\varepsilon_{v} = \varepsilon_{1} + 2\varepsilon_{3}$, the strain is positive when the volume is compressed, and the strain is negative when the volume is expanded.

It can be seen from Figs. 4, 5 and 6 that the stress–strain curves of different joint face dip Phyllites have experienced the fracture compaction, phase-elastic deformation, phase-yield phase and failure phase. In the axial compression process of Phyllite, the axial strain increases obviously in the initial compaction stage, and the lateral strain is small, mainly the crack closure inside the Phyllite sample; In the elastic deformation stage, as the axial and radial strains increase, the volumetric strain increases slowly, and the deformation of the Phyllite is mainly the elastic deformation of the mineral particles; In the yield stage, the internal cracks of the Phyllite sample are continuously generated, developed and penetrated, the transverse strain increases rapidly, and the volume strain begins to decrease; When the stress reaches the peak strength, it enters the failure phase, and the transverse strain begins to increase rapidly, eventually causing the volumetric strain increment to become negative.

Under the 0° joint angle, the stress–strain curve of the Phyllite sample is nearly straight. At the initial stage of the sample loading, the transverse strain rapidly increases, occupying the main position, and the volume expansion accompanied by the transverse strain is formed at the initial stage of loading and increases sharply. Under the 15° joint angle, the lateral strain at the initial stage of loading is small, mainly the compression along the bedding plane and the compaction of the fracture volume. Under the 30° joint angle, the sample compaction phase has a short duration, the compaction phase is not obvious, the transverse strain increases after compaction. Under the 45° joint angle, the sample compaction stage has a short duration, the transverse strain increases after compaction, and the sample extends in volume. Under the 60° joint angle, the compaction phase is obvious, and the axial strain increases rapidly after reaching the yield stage. Under the angle of 75° joint surface, the compaction process of Phyllite is remarkable. When the axial strain increases, the volumetric compression is accompanied by a large amount of axial strain, which dominates the strain process and the volumetric strain with axial strain produces large compression deformation. Under the 90° joint surface dip angle, the compaction phase of the Phyllite is very short, and the stress–strain process is linear. Before the stress reaches the peak value, the axial strain value is small, and the stress–strain curve is relatively straight. The deformation of the test piece is mainly elastic deformation, and the elastic brittle fracture occurs after reaching the peak value. At the initial stage of sample loading, the radial strain is accompanied by the axial strain, which results in a part of the axial deviatoric stress during loading. During the loading process, the fracture along the bedding direction is axially squeezed, and the transverse strain caused by the Poisson effect gradually exceeds the strain limit between the layers, so that the tensile crack propagates rapidly between the layers, which eventually lead to the separation of the sample.

Comparing the stress–strain curves of different joint dip angle Phyllites, it can be found that at α = 0°, the peak axial strain of the Phyllite is large and the axial deformation is significant. During the change of α from 0° to 45°, the axial strain begins to decrease gradually, and the axial deformation at α = 45° accounts for only 50% of α = 0°. Then, as α changes from 30° to 90°, the axial strain increases again. The peak radial deformation level of the sample is within 1%.

### Macroscopic failure mode and intensity analysis

**Figure 7:**
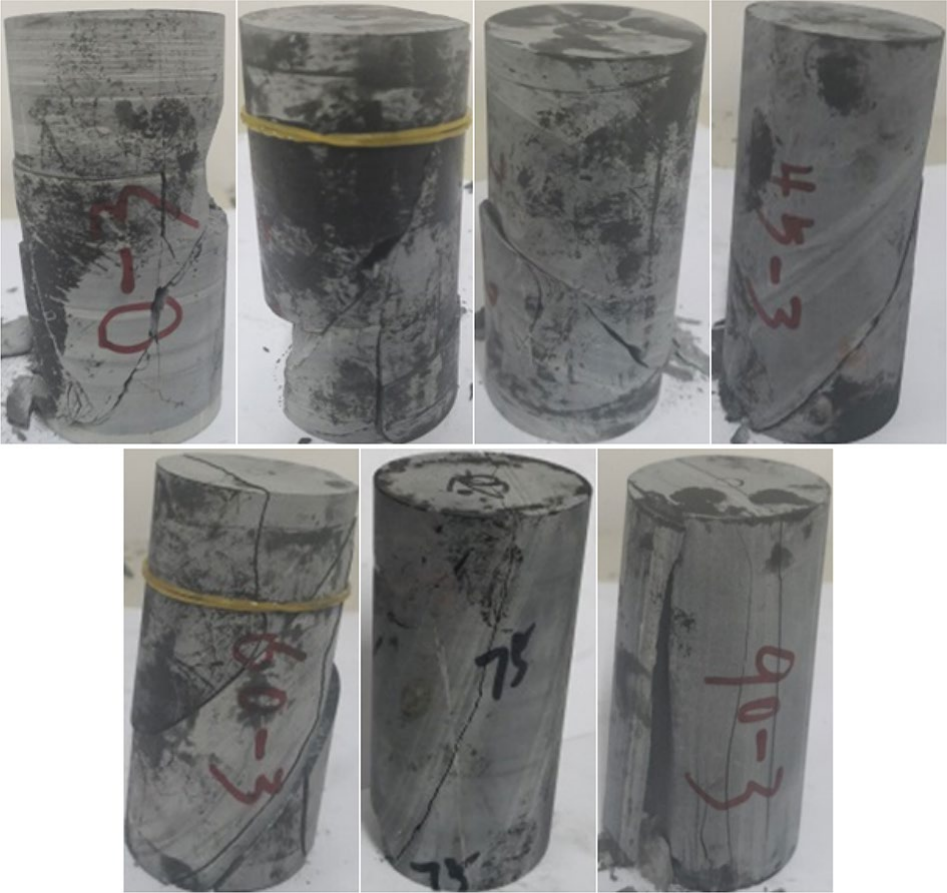
Macroscopic destruction picture of Phyllite.

The failure mode of different joint face dip Phyllite is shown in Fig. 7. It can be seen from Fig. 7 that the macroscopic failure modes of Phyllite can be mainly divided into shear failure, shear slip and tensile failure, and separation tension failure.

When α = 0°, the failure mode of Phyllite is shear failure. The crack is mainly a shearing main crack, and there is vertical tensile crack along the main crack direction. It is connected with the main crack, causing the test block to break and fall, and the periphery of the sample is accompanied by the peeling phenomenon. When α = 15°, the failure mode of phyllite is shear failure. The crack is mainly a large penetrating crack along the diagonal direction of the sample, accompanied by a vertical tensile crack along the main crack direction, which penetrates the main crack and causes the test block to peel off. When α = 30°, the failure mode of phyllite is shear slip tension failure. The crack is mainly a shear through crack. The end of the crack is subjected to tensile stress, which makes the end of the crack extend vertically, and there is a small amount of crushing phenomenon at the end of the sample. When α = 45°, the failure mode of Phyllite is shear failure along the bedding plane. The crack is mainly a through-type crack along the bedding plane, and there is a small amount of crushing at the end of the sample. When α = 60°, the failure mode of Phyllite is shear slip and tensile composite failure. The cracks are mainly two shear-slip cracks along the bedding plane and tensile cracks in the vertical direction. The sample forms a plurality of through-fracture surfaces and slides along the direction of the layered surface, accompanied by the breakage of the tensioned blocks. When α = 75°, the failure mode of Phyllite is the shear failure along the bedding plane. The crack is mainly a through-type crack in the direction of one laminar plane, and a small-scale crushing and cracking block appears at the corners, the sample is relatively complete. When α = 90°, the failure mode of Phyllite is the tensile failure of the layer. The sample has a tensile and brittle failure phenomenon which is mainly parallel in the axial direction and is mainly caused by pressure-induced tensile cracking. The cracks are basically parallel to the axial direction, and there are many through-type cracks along the axial direction. The sample formed a complete failure surface accompanied by shedding of the surface portion of the surface.

Comparing the failure modes, it can be found that when 0 ≤ α ≤ 15°, the phyllite sample mainly undergoes shear failure, and when 30° ≤ α ≤ 75°, the main shear slip tension along the bedding plane occurs. Failure, the inclination of the bedding surface controls the failure surface form of the phyllite. When α > 75°, the phyllite sample will fail under tension. As the inclination of the joint plane increases, the failure mode of the phyllite gradually changes from shear failure to tension failure.

**Figure 8:**
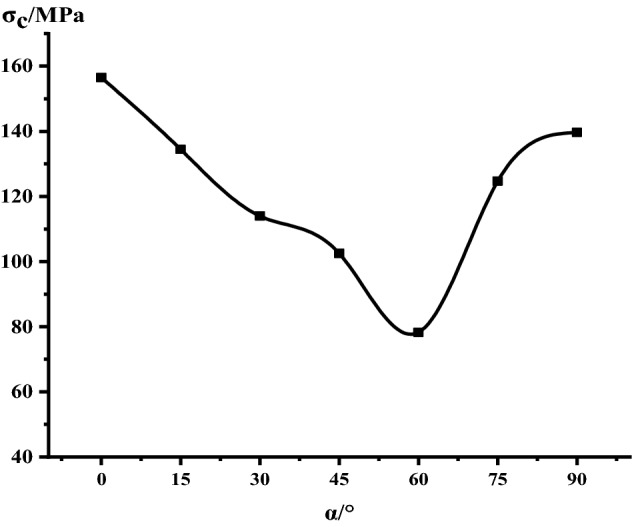
Uniaxial peak compressive strength as a function of joint angle.

Figure 8 shows the Uniaxial peak compressive strength as a function of joint angle. The anisotropy of the strength of the Phyllite is significant, with a “U” shape change with the dip angle of the joint. As the angle of the joint surface increases, the uniaxial compressive strength of the Phyllite changes significantly. When α = 0°, the uniaxial compressive strength of the Phyllite reaches the maximum value, reaching 156.49 MPa. With the increase of the dip angle α of the joint surface, the strength gradually decreases. When α = 60°, the compressive strength achieved a minimum of only 76.25 MPa, and the ratio of the maximum to the minimum uniaxial compressive strength was 2.05.

### Analysis of progressive failure process of phyllite

**Figure 9:**
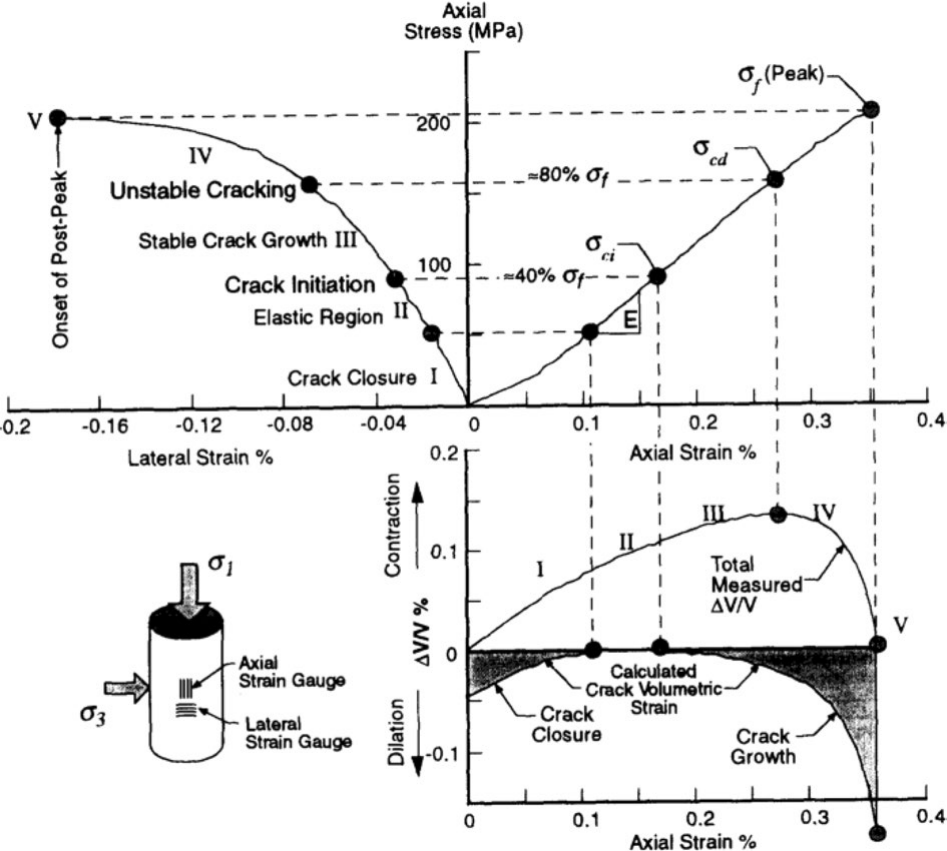
Schematic diagram of gradual failure stage (Martin 1994).

**Figure 10:**
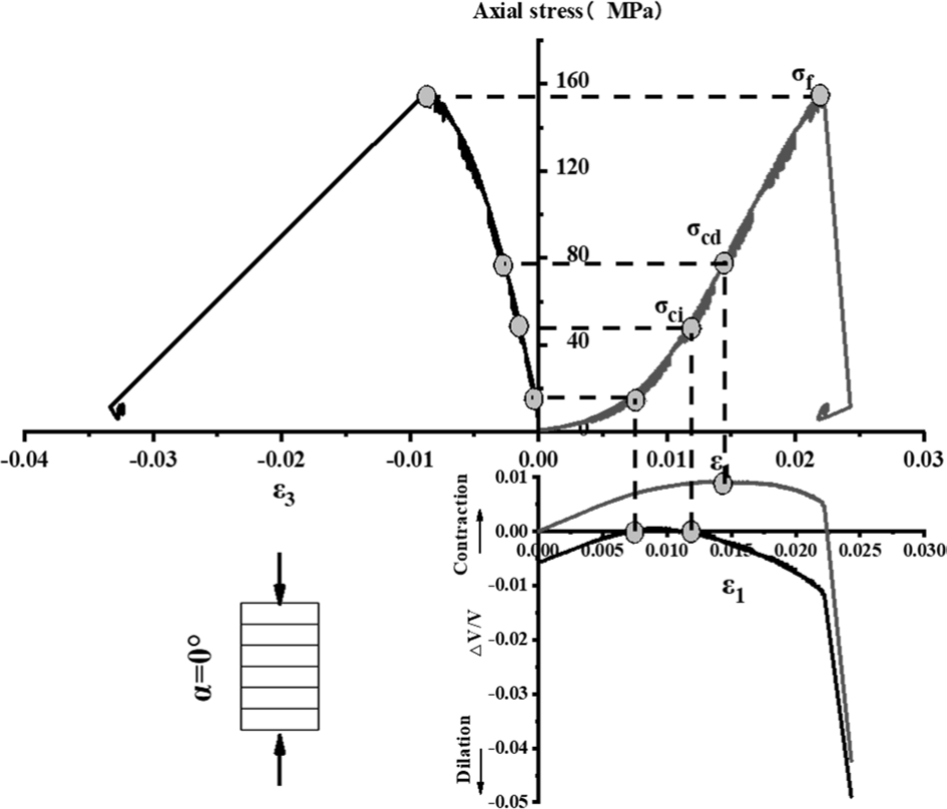
Progressive failure curve when the joint plane is α = 0°.

**Figure 11:**
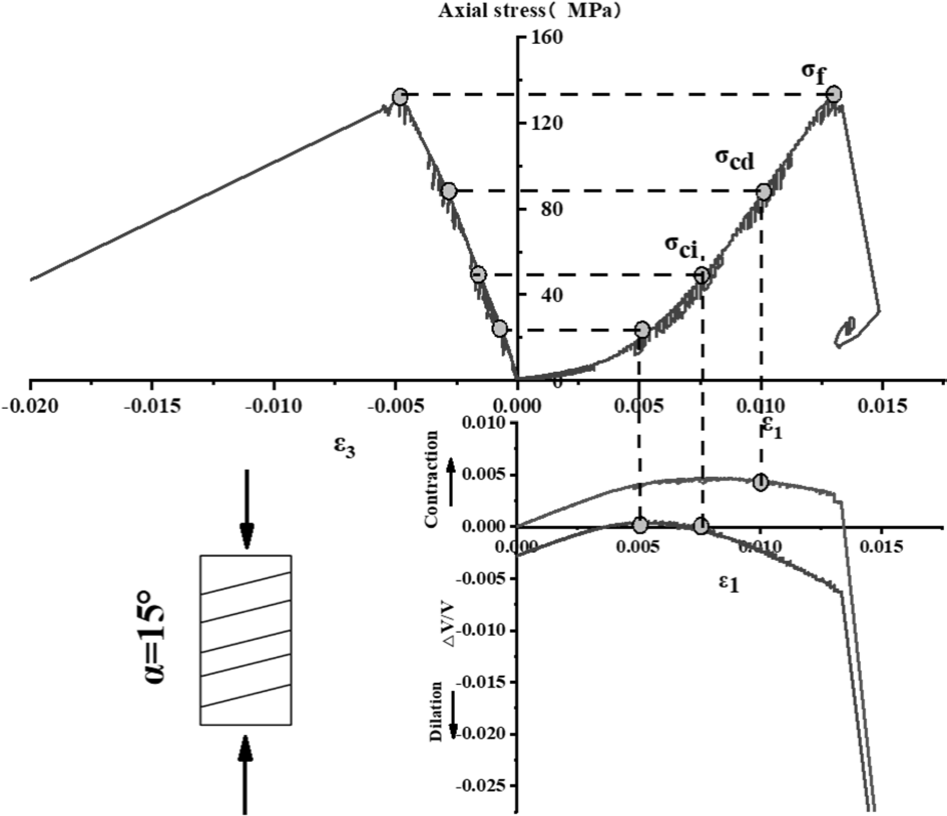
Progressive failure curve when the joint plane is α = 15°.

**Figure 12:**
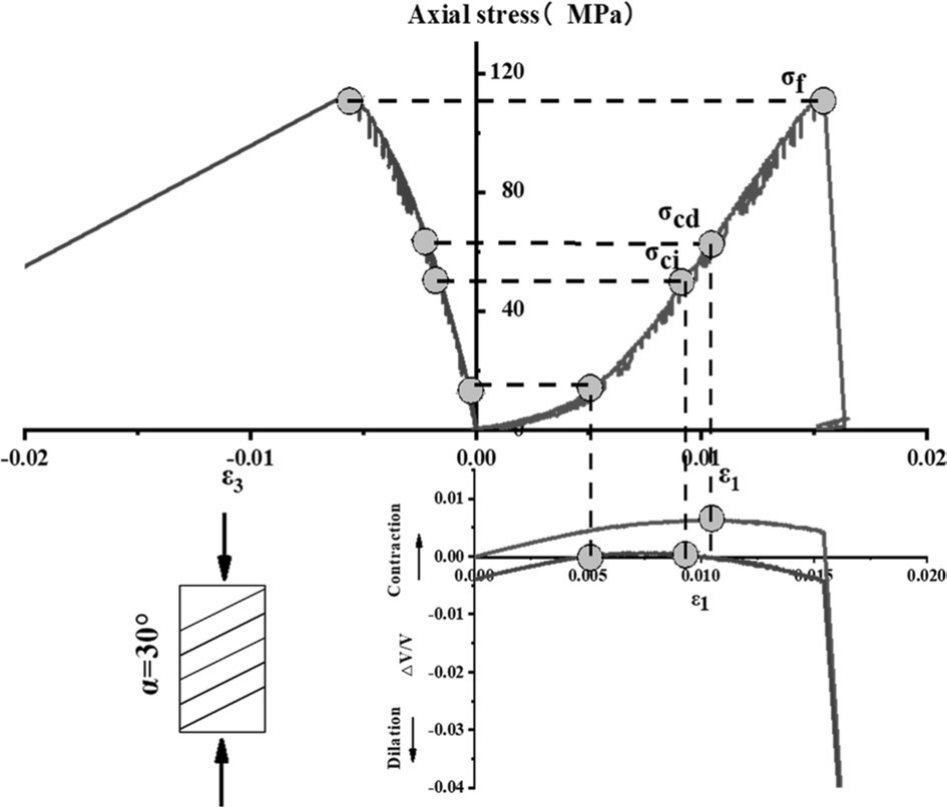
Progressive failure curve when the joint plane is α = 30°.

**Figure 13:**
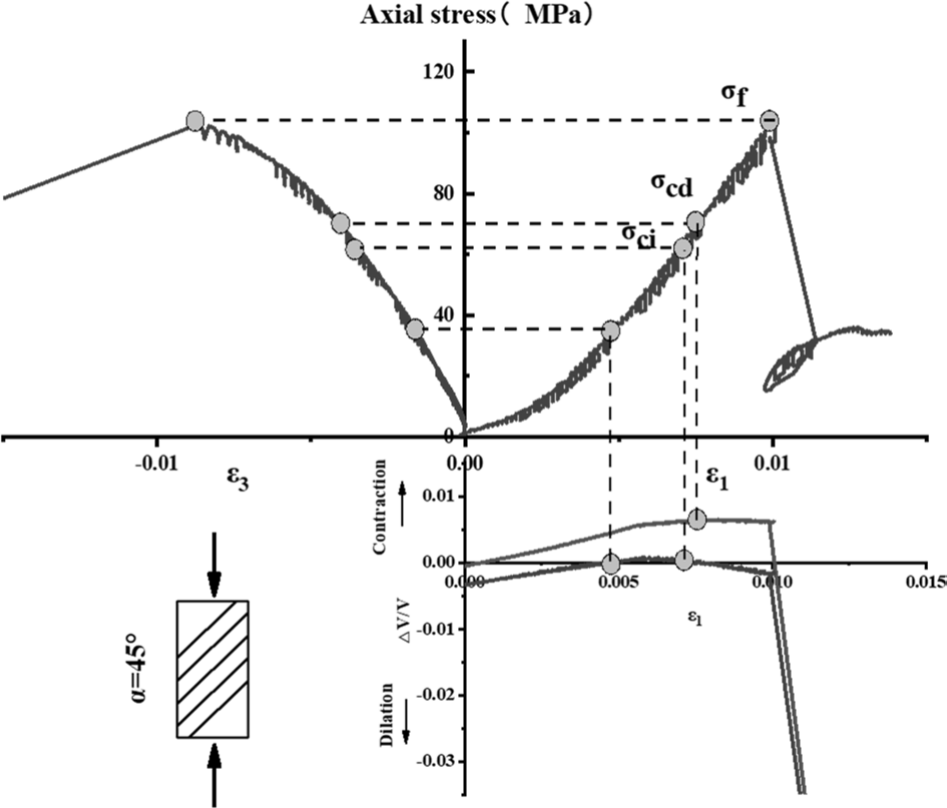
Progressive failure curve when the joint plane is α = 45°.

**Figure 14:**
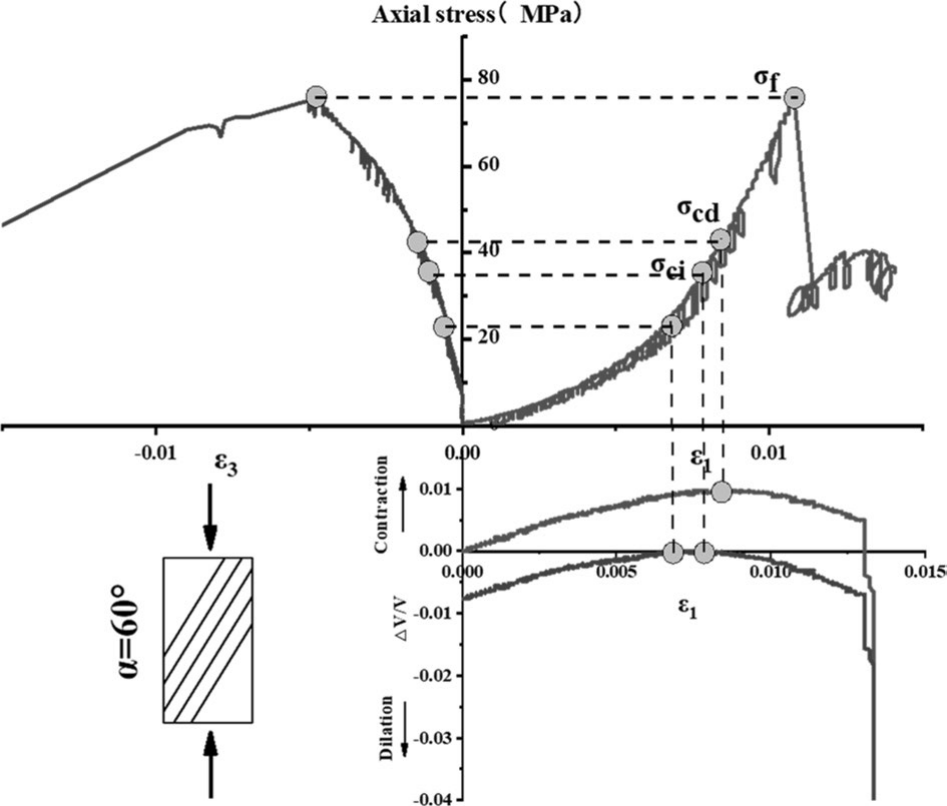
Progressive failure curve when the joint plane is α = 60°.

**Figure 15:**
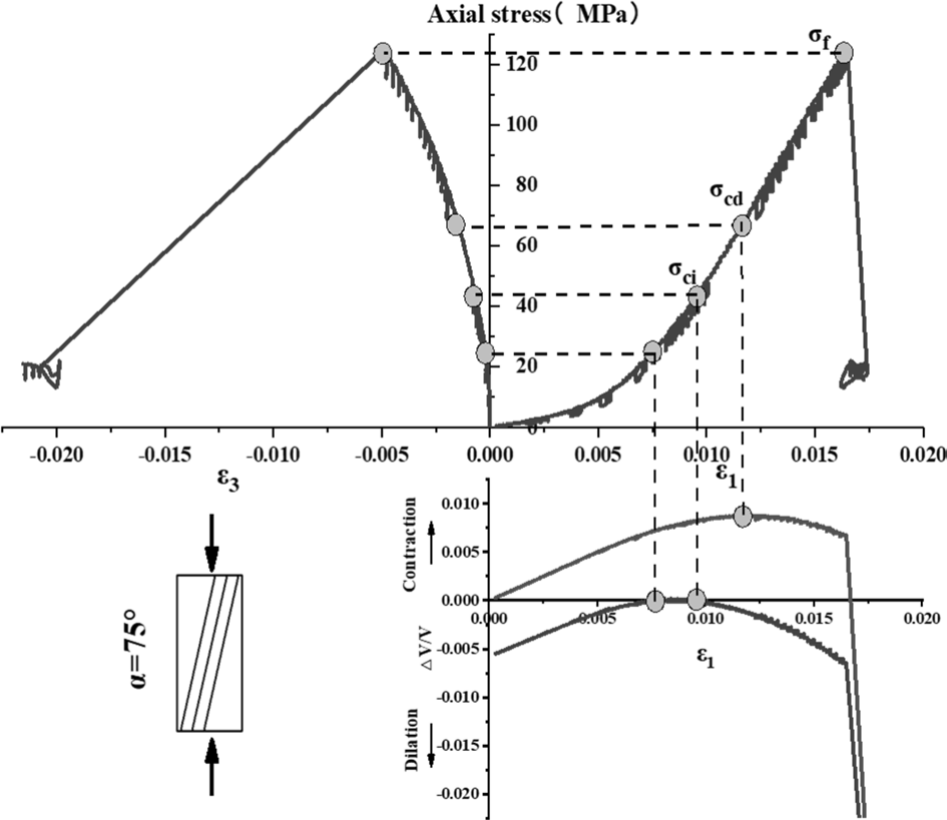
Progressive failure curve when the joint plane is α = 75°.

**Figure 16:**
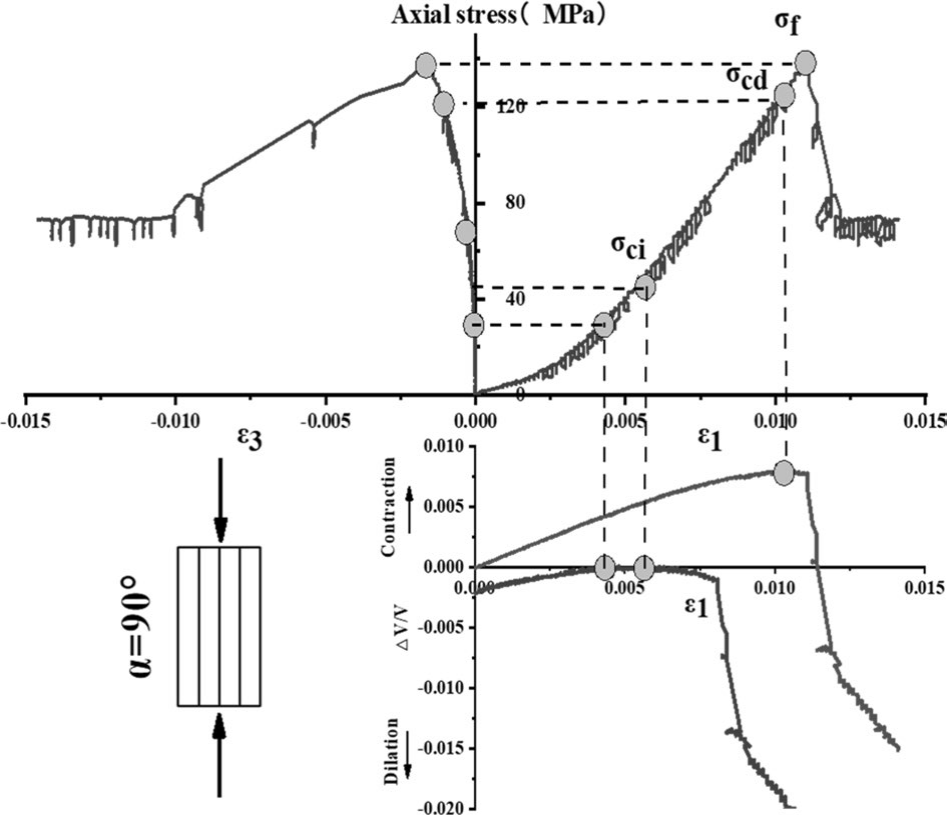
Progressive failure curve when the joint plane is α = 90°.

The stress–strain curve of Phyllite can be divided into five zones (as shown in Fig. 9). The I partition is the stage where the crack has been closed in the sample. Once the crack has been closed in the sample, the material at this time can be assumed to be a linear elastic and isotropic material (II partition). The fracture volume strain just begins to increase at the beginning of the axial stress mark III, and the starting point of the IV partition corresponds to the inverse bend point of the sample volume stress–strain curve, representing the beginning of unstable crack propagation. The post-peak stage corresponds to the V partition. Figures 10, 11, 12, 13, 14, 15 and 16 shows the uniaxial compression fracture process of phyllite at different inclinations.

The total volumetric strain is calculated from the axial strain and the transverse strain:

1 $${\upvarepsilon }_{{\text{v}}} = \frac{{\Delta {\text{V}}}}{{\text{V}}} \approx {\upvarepsilon }_{1} + 2{\upvarepsilon }_{3} .$$

The elastic volumetric strain is calculated by the following formula:

2 $${\upvarepsilon }_{{{\text{ve}}}} = \frac{{\Delta {\text{V}}}}{{{\text{V}}_{{{\text{elastic}}}} }} = \frac{{1 - 2{\upmu }}}{{\text{E}}}{(}{\upsigma }_{1} - {\upsigma }_{3} {)}{.}$$

The fracture volumetric strain is obtained by subtracting the elastic volumetric strain from the total volumetric strain:

3 $${\upvarepsilon }_{{{\text{vc}}}} = {\upvarepsilon }_{{\text{v}}} - {\upvarepsilon }_{{{\text{ve}}}} .$$

**Figure 17:**
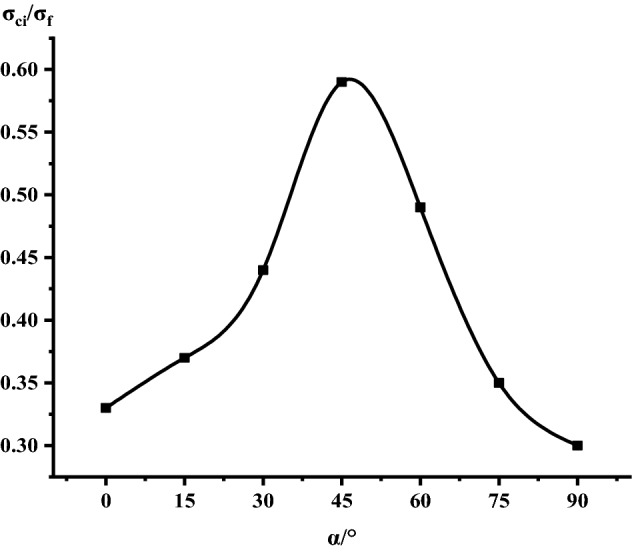
σ_ci_/σ_f_ Curve with the dip angle of the joint.

**Figure 18:**
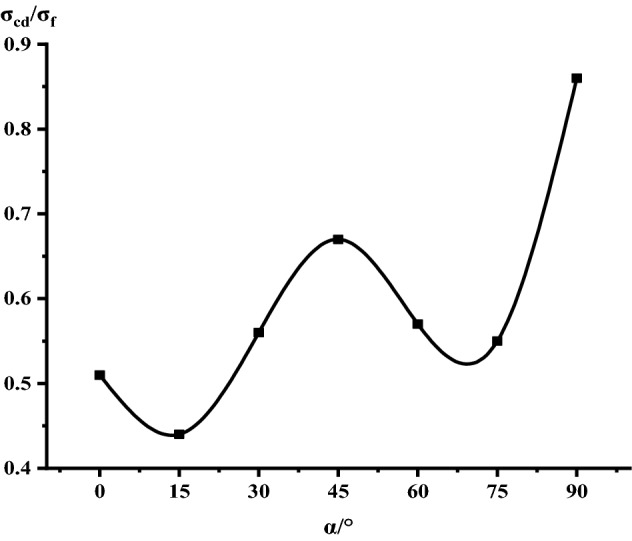
σ_cd_/σ_f_ Curve with the dip angle of the joint.

It can be seen from Table 1, Figs. 17 and 18 that in the process of α increasing from 0° to 45°, σ_ci_/σ_f_ gradually rises, the crack initiation stress appears later, and the unstable development stage of crack becomes shorter. In the process of α continuing to grow to 90°, σ_ci_/σ_f_ starts to gradually decrease again, and at α = 90°, σci/σf appears to be the minimum. This is related to the internal structure of the Phyllite, which is a weak surface with tensile strength. When the parallel layer is loaded, it will be pulled in the vertical direction, and at the lower stress level, the crack will occur. When the crack increases, the crack combines and penetrates until the crack breaks the stress. Therefore, when α = 90°, the crack initiation stress σ_ci_ appears earlier in the loading process, and the crack unstable development stage is longer. When the dip angle α of the joint surface changes from 90° to 45°, the angle between the loading direction and the plane of the bedding plane increases gradually, the effective force between the layers decreases continuously, and the crack initiation stress level (σ_ci_/σ_f_) increases continuously. It is broken for sliding or shearing along the joint surface. During the change of α to 0°, the failure mode begins to change from shear slip along the joint surface to shear failure. The micro-cracks inside the Phyllite begin to appear at the beginning of loading, and σ_ci_/σ_f_ decreases.

**Table 1:** Threshold value of dip angle stress of different joints of Phyllite.

Stress threshold	α/°						
	0°	15°	30°	45°	60°	75°	90°
σ_ci_(MPa)	50.86	49.98	50.31	60.86	37.44	43.51	42.29
σ_cd_(MPa)	79.68	59.46	63.79	68.71	43.12	68.98	120.72
σ_f_(MPa)	156.49	134.47	113.97	102.55	76.25	124.69	139.64
σ_ci_/σ_f_	0.33	0.37	0.44	0.59	0.49	0.35	0.30
σ_cd_/σ_f_	0.51	0.44	0.56	0.67	0.57	0.55	0.86

The crack initiation stress level of different joint dip angle Phyllite is 0.30–0.59σf, and the crack failure stress level is 0.44–0.86σf. When the axial force exceeds the crack failure stress, the crack grows rapidly and unstable until it breaks. When α = 45°, the value of σ_ci_/σ_f_ is the largest, and the initial stress level of crack is later. Combined with the macroscopic failure mode of Phyllite, it can be seen that the failure is the shear failure along the joint surface, and the stress at the initial stage of loading increases. After reaching the stress threshold of the joint surface failure, the crack rapidly generates and extends to the damage. When α = 90°, the value of σ_cd_/σ_f_ is the largest, and the reliable peak intensity of rock is the largest, which is 120.72 MPa.

## Discussion on fracture mechanism

**Figure 19:**
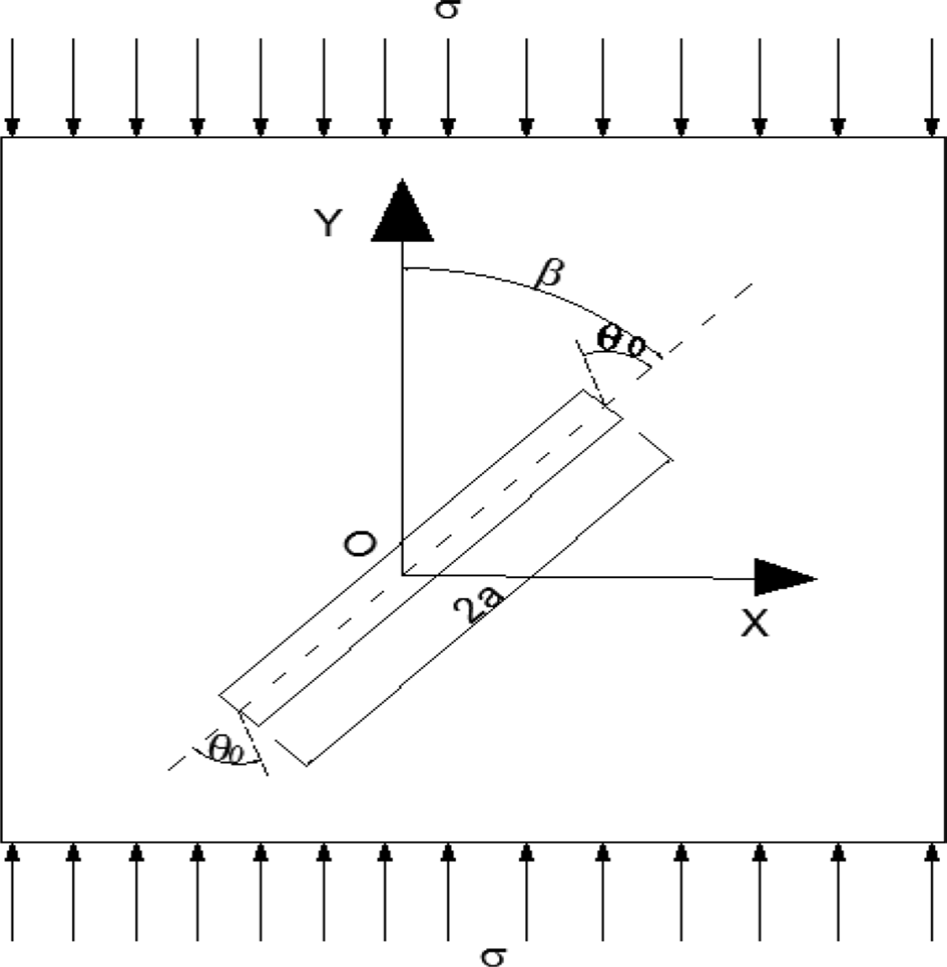
Schematic diagram of a single microcrack subjected to uniaxial compression load.

For a long time, the problem of rock fracture mechanism has attracted much attention. Many scholars have carried out a series of experiments on various rocks, such as Aliha et al.^27–29^ uses a new type of cracked disc specimen to experimentally study the I/III mixed fracture toughness of a typical rock material under bending load. It was shown that the specimen can provide full combinations of modes I and III and consequently a complete set of mixed mode I/III fracture toughness data were determined for the tested marble rock. And uses several cracked zigzag-notched Brazilian discs (CCNBD) specimens to experimentally study the fracture toughness of white marble under pure type I and pure type II loading, using the generalized maximum tangential stress theory, according to I The type fracture toughness data estimates the obtained type II test results. proposed that mixed mode fracture prediction does not need to directly determine the crack tip fracture parameters, but considers the total tangential stress component in predicting the corresponding fracture load in any desired mode I/II mixture. The TSC criterion is used to predict the mixed type I/II fracture load of the edge crack asymmetric four-point bending (AFPB) sample of granite and marble. Akbardoost et al.^30^ uses two kinds of specimens: edge cracked semicircular bend (SCB) under the action of three-point bending and cracked Brazilian disc (BD) under the action of diameter compression. Different modal mixed tests from pure type I to pure type II were carried out for each type of specimen, and the tests were carried out on specimens of different sizes characterized by 4 specimen radii of 25 to 150 mm. The experimental results show that the mixed-mode fracture resistance increases with the increase of sample size. The modified maximum tangential stress criterion (MMTS) is used to predict the f racture resistance of Guiting limestone under mixed loads. In this paper, the development of single microcrack under uniaxial compression load is taken to study the failure mechanism of rock under uniaxial compression load. At the center of the rock of a unit thickness is a central crack of 2a length and Angle β to the direction of load. The axial load is σ (as shown in Fig. 19).

By fracture mechanics, the stress intensity factor of rock under pure type I loading is $K_{I} = \sigma \sqrt {\pi a}$. The stress intensity factor under pure type II loading is $K_{II} = \tau \sqrt {\pi a}$. Under uniaxial compression, the rock stress type is I–II type composite loading, the stress intensity factor of rock are as follows:

4 $$K_{I} = - \sigma \sqrt {\pi a} \sin^{2} \beta ,$$

5 $$K_{II} = - \sigma \sqrt {\pi a} \sin \beta \cos \beta .$$

In the formula, the positive direction of σ is taken as the direction of tensile stress.

There are many theoretical criteria for rock failure, such as the maximum circumferential tensile stress criterion, which considers that the crack propagation is along the section with the maximum circumferential tensile stress σ_θmax_, and the condition of crack propagation is when the stress intensity factor reaches a critical value, which is the fracture toughness of the material. The strain energy density factor fracture criterion is proposed by considering the effect of six stress components near the crack tip, the local strain energy density near the crack tip is calculated, and the local strain energy density is compared on the concentric circles with the crack tip as the center. According to the theory of maximum energy release rate, the crack propagates along the direction of the maximum energy release rate, and the crack begins to propagate when the maximum energy release rate reaches a critical value. According to the theory of maximum tensile stress, the direction of crack propagation is perpendicular to the stress axis at the crack tip. When the tensile stress at the crack tip reaches a critical value, the crack begins to expand. In this paper, the maximum circumferential stress theory is adopted to study the fracture of rock under uniaxial compression. In polar coordinates, the stress component of rock crack tip is shown as follows^31^:

6 $$\sigma_{r} = \frac{1}{{2\sqrt {2\pi r} }}\left[ {K_{I} \cos \frac{\theta }{2}\left( {3 - \cos \theta } \right) + K_{II} \sin \frac{\theta }{2}\left( {3\cos \theta - 1} \right)} \right],$$

7 $$\sigma_{\theta } = \frac{1}{{2\sqrt {2\pi r} }}\cos \frac{\theta }{2}\left[ {K_{I} \left( {1 + \cos \theta } \right) - 3K_{II} \sin \theta } \right],$$

8 $$\tau_{r\theta } = \frac{1}{{2\sqrt {2\pi r} }}\cos \frac{\theta }{2}\left[ {K_{I} + \sin \theta + K_{II} \left( {3\cos \theta - 1} \right)} \right].$$

In the formula, the symbol $\theta_{0}$ is the fracture Angle, representing the included Angle between the fracture section and the original crack line. According to the maximum circumferential stress theory, the fracture Angle of the rock meets the following conditions:

9 $$\frac{{\partial \sigma_{\theta } }}{\partial \theta } = 0,$$

10 $$\frac{{\partial^{2} \sigma_{\theta } }}{{\partial \theta^{2} }} < 0.$$

Combining Eqs. (6)–(8), the fracture Angle of the rock is obtained as follows:

11 $$\frac{{1 - 3\cos \theta_{0} }}{{\sin \theta_{0} }} = \tan \beta .$$

Fracture Angle of the type that is associated with the Angle of crack and loading direction, Under type I–II composite loading, the crack will begin along the direction of the $\theta_{0}$ extension, rather than on the direction along the crack line. At this point, the maximum circumferential stress is:

12 $$\sigma_{\max } = \frac{1}{{2\sqrt {2\pi r} }}\cos \frac{{\theta_{0} }}{2}\left[ { - \sigma \sqrt {\pi a} \sin^{2} \beta \left( {1 + \cos \theta_{0} } \right) + 3\sigma \sqrt {\pi a} \sin \beta \cos \beta \sin \theta_{0} } \right].$$

By above knowable, when rock under uniaxial compression condition, the micro cracks in I–II complex stress state, the crack is not along with the original crack direction continues to expand, but when the stress intensity factor reaches the fracture toughness of the rock, the crack along with the original crack line into $\theta_{0}$ direction begin to expand.

## Uniaxial compression simulation of Phyllite based on RFPA2D

The RFPA analysis system is based on the finite element theory and the new material rupture algorithm idea. It uses elastic mechanics as the basis for stress analysis, and elastic damage theory as the basis for medium deformation. It simulates the nonlinearity of the force by considering the non-uniformity of the material. The weakening of the element is used to simulate material deformation and failure, which has a good simulation effect for studying the process of rock mass from mesoscopic failure to macroscopic failure.

### Model establishment

**Figure 20:**
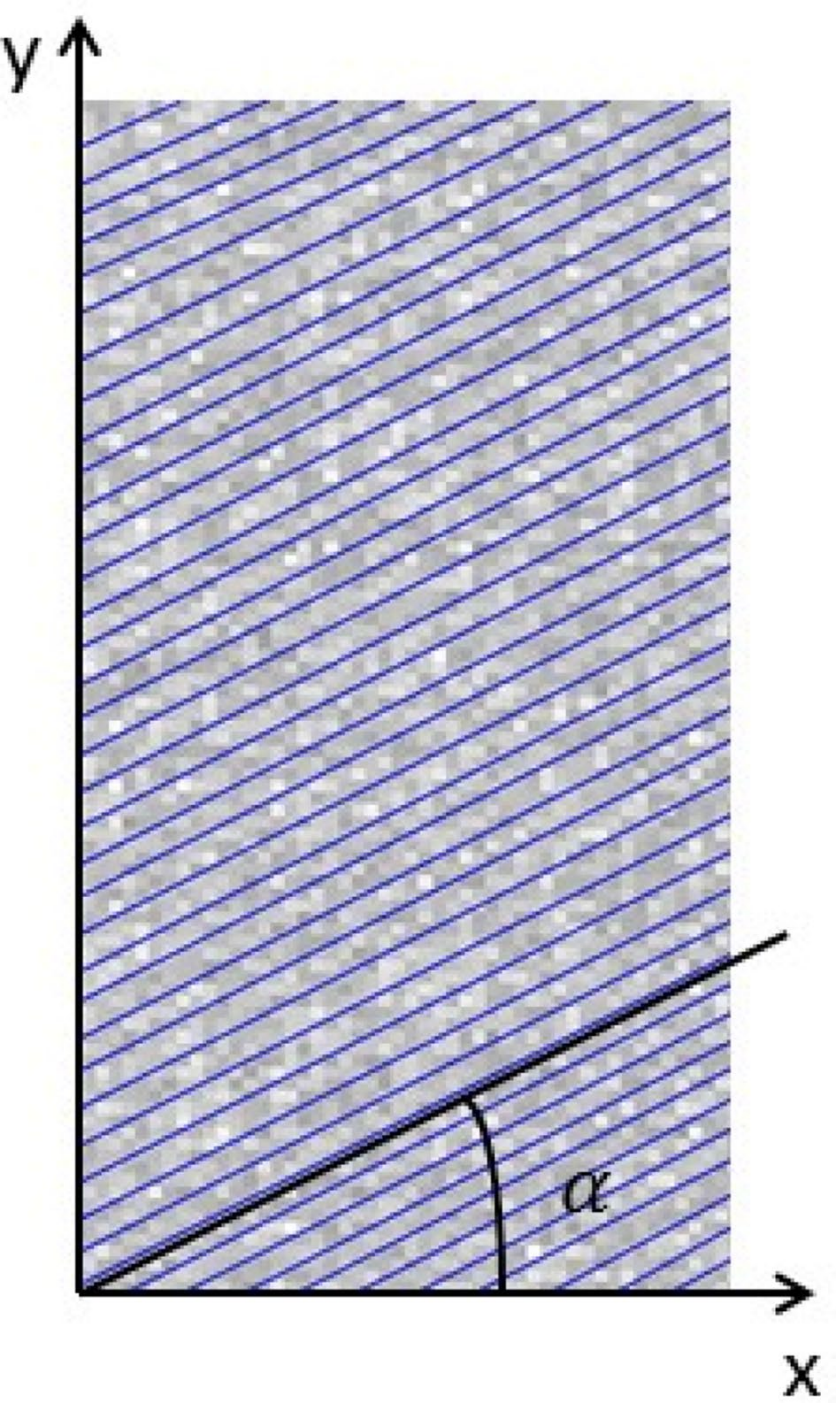
Schematic diagram of phyllite model.

In order to study the Joint phyllite gradual damage under uniaxial compression, based on the RFPA2D analysis system to simulate the bedding direction respectively 0°, 15°, 30°, 45°, 60°, 75°, 90° phyllite failure process, It is through RFPA2D—Basic analysis system to establish numerical model in Fig. 19, the model unit color grayscale representation unit size of the modulus of elasticity, the light grey area of the elastic modulus is larger, Fig. 20 units for phyllite matrix of gray, The blue unit represents the Phyllite joint surface, Where α represents the angle between the Joint surface and the horizontal axis.

The model is a cylindrical sample (diameter: 50 mm, height: 100 mm), the mesh is divided into 50 × 100, the loading method is displacement loading control, and the loading step is 0.002 mm/step. In the model, the elastic modulus and strength of the phyllite sample obey Weibull random distribution. The initial microscopic parameters of the phyllite sample are shown in Table 2 below.

**Table 2:** Mesoscopic parameters of phyllite model.

material	Homogeneous degree (m)	Modulus of elasticity E (MPa)	Uniaxial compressive strength ${ }\sigma_{c}$ (MPa)	Poisson’s ratio ν	Friction angle (°)	Pressure rabbi	Percentage of residual strength (%)
Phyllite matrix	5	50,000	500	0.3	30	10	0.1
Joint surface	2	30,000	420	0.4	28	10	0.1

### Simulation results and analysis

**Figure 21:**
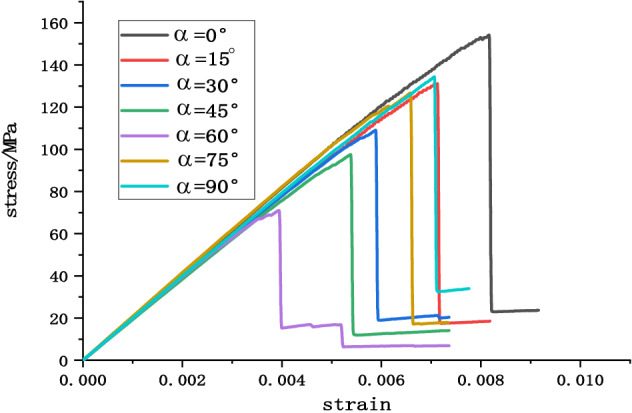
Stress–strain curves of phyllite under different bedding dips.

**Figure 22:**
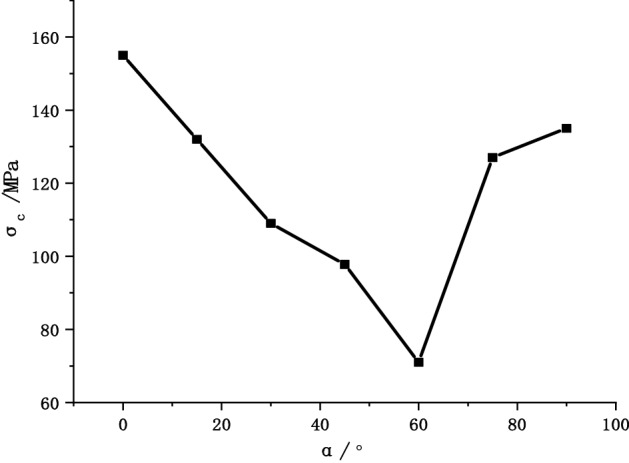
Peak stress-bedding inclinations curve.

Figure 21 shows the simulated stress–strain curves of phyllite under different joint inclinations, the figure shows that the stress–strain curve of phyllite are greatly influenced by the Joint inclination, But generally it still shows elasticity, yielding and failure stages, In the initial stage, there is no obvious crack compaction stage. In the elastic stage, the stress–strain relationship becomes linear. In the yield stage, the growth rate of the stress–strain curve gradually decreases. After the peak stress is exceeded, the stress decreases linearly. And finally stabilized, showing strong brittleness. It can be seen from Fig. 22 that the peak stress of phyllite changes significantly with the joint inclination change, showing strong anisotropy characteristics. The overall trend of change is that as the joint inclinations increases, the peak stress first decreases and then rises. U”-shaped change, which is consistent with the results of the indoor test. When α = 0°, the uniaxial compressive strength of phyllite achieves the maximum value, and when α = 60°, the uniaxial compressive strength of phyllite obtains the minimum value.

In order to express the influence of joint direction on the uniaxial compressive strength of phyllite, the joint effect coefficient D(α) is proposed to characterize the joint structure effect, and its expression is as follows:

13 $${\text{D}}\left( \alpha \right) = 1 - \frac{{\sigma_{\alpha } }}{{\sigma_{0} }}.$$

In the formula, $\sigma_{\alpha }$ represents the uniaxial compressive strength of the rock when the joint inclinations is α, and $\sigma_{0}$ represents the uniaxial compressive strength of the rock when the joint inclinations is 0°.

Substituting the results of the indoor test and simulation into the above formula, the results of the joint effect coefficient of the phyllite indoor test and simulation (shown in Table 3) are obtained. It can be seen from Table 3 that when α = 60°, the experimental value joint effect coefficient is 0.51, and the simulated value joint effect coefficient is 0.54. When the joint inclinations is 60°, the joint structure effect of phyllite is the strongest. As the joint inclinations increases, the joint effect coefficient shows a trend of first increasing and then decreasing. The larger the value of the joint effect coefficient, the greater the influence of the joint inclinations on the compressive strength of the rock.

**Table 3:** Table of bedding effect coefficients of uniaxial compressive strength of phyllite.

Joint inclinations (°)	Experimental value		Simulation value	
	Compressive strength (MPa)	Joint effect coefficient D(α)	Compressive strength (MPa)	Joint effect coefficient D(α)
0	156.49	0	154.86	0
15	134.47	0.14	131.65	0.15
30	113.97	0.27	108.92	0.30
45	102.55	0.34	97.83	0.37
60	76.25	0.51	71.63	0.54
75	124.69	0.20	127.35	0.18
90	139.64	0.11	135.28	0.13

**Figure 23:**
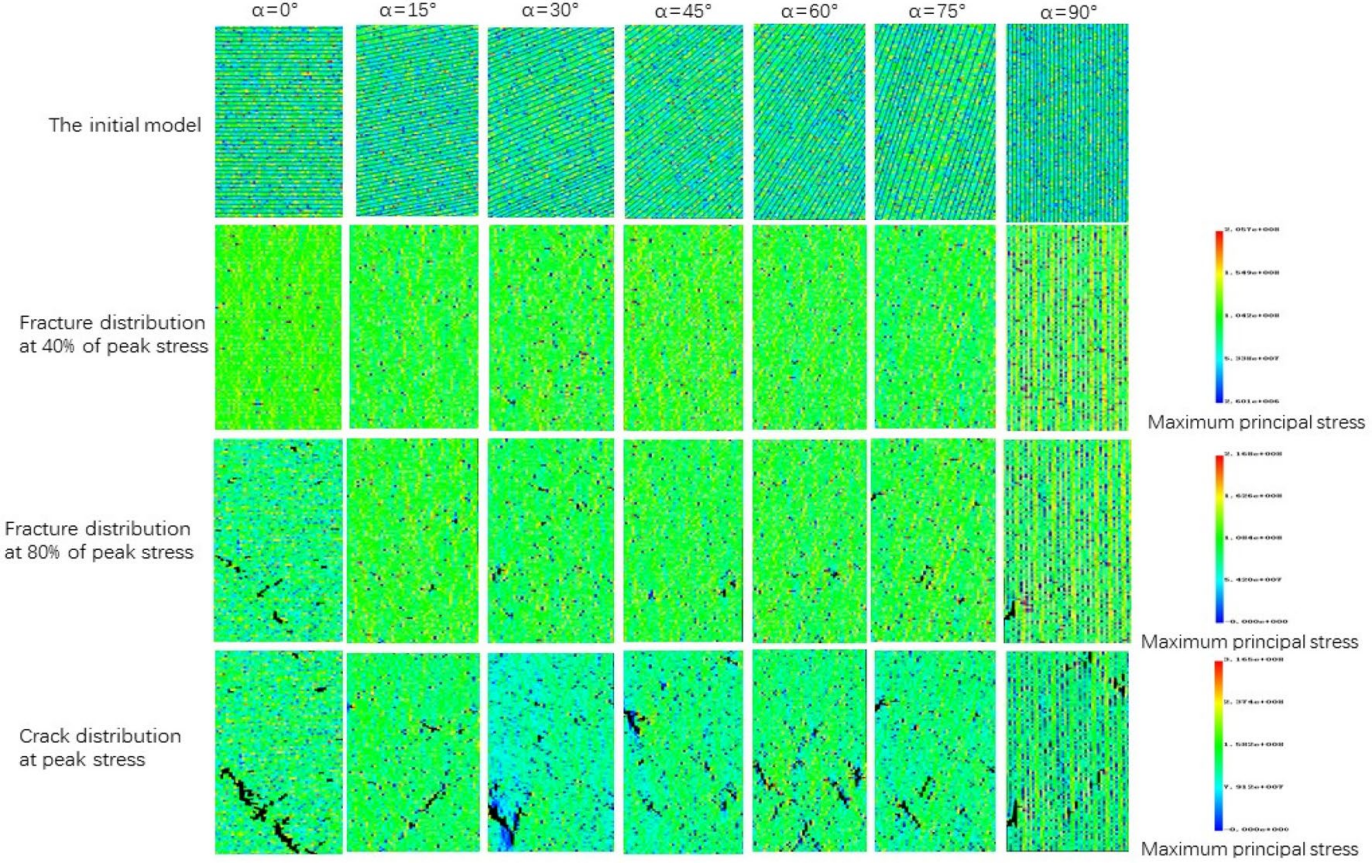
Schematic diagram of progressive destruction of Phyllite under different joint inclinations.

**Figure 24:**
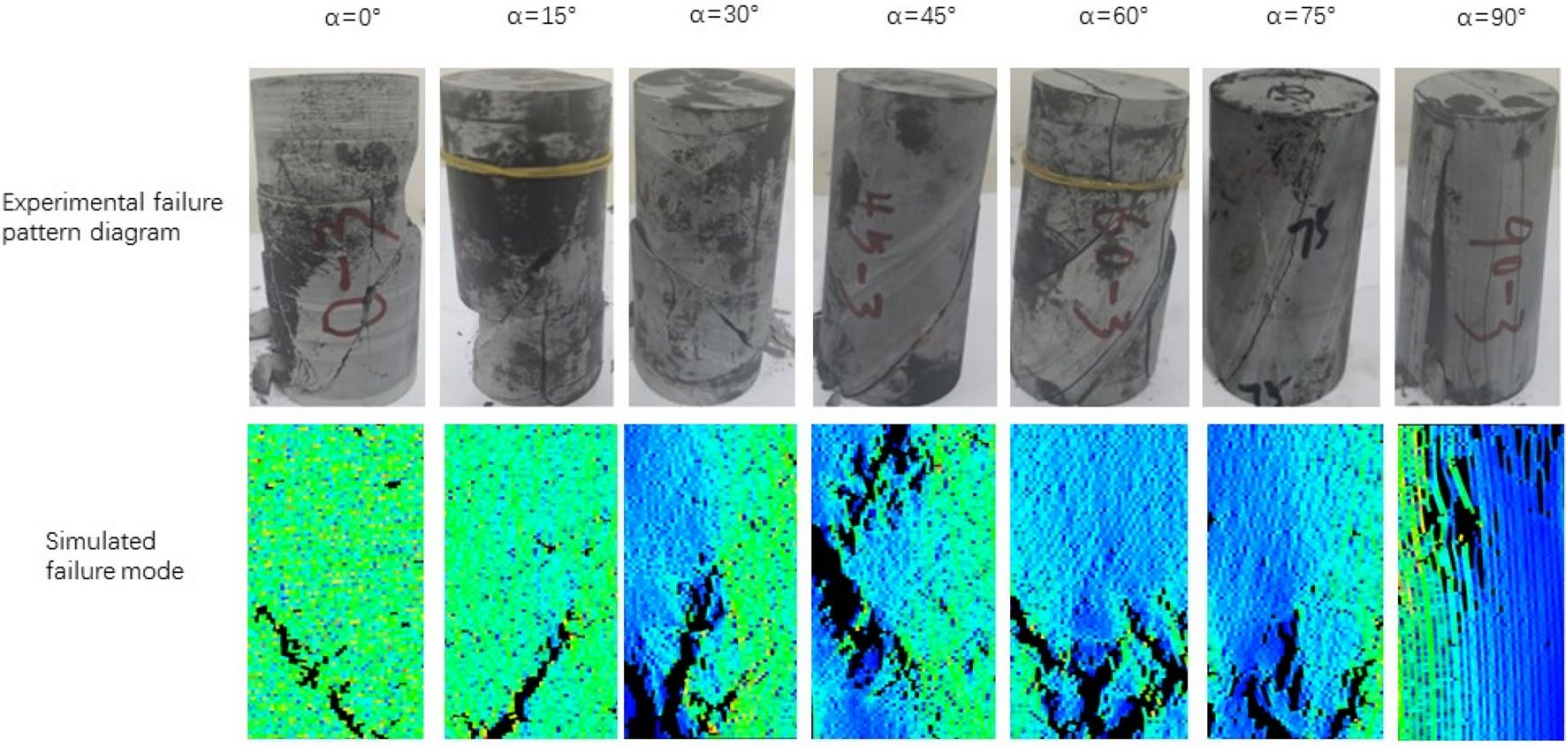
Comparison of phyllite uniaxial compression test and simulated failure mode.

Figure 23 is a schematic diagram of progressive fracture process obtained by simulating uniaxial compression failure of Phyllite under different joint inclinations. It can be seen from the figure that the failure mode of phyllite is greatly affected by the joint direction, and can be divided into three types according to the failure mode. (A) Shear failure, when the 0° ≤  α ≤ 15°. Under the action of the load, the phyllite first appeared small cracks in the lower strength position, and then the cracks continued to extend and expand under the action of external force, Its extension direction is not along the direction of the Joint direction, but forms a certain angle with the Joint direction, and finally a slanted crack is formed and the rock is destroyed. (B) Tensile and shear damage (30° ≤ α  ≤ 75°). The initial cracks began to appear in the local area of phyllite, and the cracks showed a tendency to expand along the joint direction under load. In addition, there is a strong cementation between the stratified phyllite matrix and the joint surface, so the surrounding minerals are affected by a tensile force during the crack extension stage, and finally show tensile and shear failure. (C) The dissociation tension failure (90°), when = 90°, the initial fracture appears at the top of the rock, and then the fracture extends along the joint direction under the action of external load, showing the dissociation tension failure. According to Fig. 24, comparing the failure mode of phyllite uniaxial compression test and simulation, it is found that the failure mode basically conforms to the above-mentioned failure law, which fully verifies the correctness of the proposed uniaxial compression failure mode of phyllite.

## Conclusions

Based on phyllite as the research object, this paper adopts the indoor test, the method of combining the theoretical analysis and numerical simulation, illustrates the different joints under the stress–strain curve difference of phyllite, analyses the change of the joint plane Angle influence on phyllite failure mode, are analyzed from the points of view of fracture mechanics of phyllite under uniaxial compression fracture extension, using comparative analysis, numerical simulation and experimental results validate the joint under uniaxial compression mechanics characteristics of phyllite, macroscopic failure mode and crack development situation. The specific results are as follows:

1. The anisotropy of the uniaxial compressive strength of the jointed Phyllite is significant, and it changes with the “U” shape of the joint surface. When α = 0°, the uniaxial compressive strength of Phyllite reaches a maximum of 156.49 MPa, and the strength gradually decreases during the process of α increasing from 0° to 60°. When α = 60°, the uniaxial compressive strength achieved a minimum value of 76.25 MPa. The ratio of the maximum to the minimum uniaxial compressive strength was 2.05.
2. The macroscopic failure forms of phyllite can be divided into three types: shear failure, shear-slip combined tension failure and separation layer tension failure. When 0 ≤ α ≤ 15°, the phyllite sample mainly undergoes shear failure, and when 30° ≤ α ≤ 75°, shear slip and tension failure along the bedding plane mainly occur, and the bedding plane dip angle is controlled In view of the failure surface form of phyllite, when α > 75°, the phyllite sample will fail under tension.
3. The crack initiation stress level of the joint Phyllite is 0.30–0.59σ_f_, and the crack failure stress level is 0.44–0.86σ_f_. When α = 90°, the crack initiation stress σ_ci_ appears earlier in the loading process, the value of σ_cd_/σ_f_ is the largest, and the stage of unstable crack development is longer. The σ_cd_ of α = 90° can be used as the maximum reliable value of the uniaxial compressive strength of the Phyllite.
4. The joint structure effect of phyllite is the strongest when the Joint inclination is 60°. As the Joint inclination increases, the joint effect coefficient shows a trend of first increasing and then decreasing. Among them, the greater thejoint effect coefficient value. Indicating that the Joint inclination has a greater impact on the compressive strength of the rock.

## Abbreviations

α: Phyllite joint dip angle
$$\sigma_{c}$$: Axial stress
$$\varepsilon_{1}$$: Axial strain
$$\varepsilon_{3}$$: Horizontal strain
$$\varepsilon_{v}$$: Volume strain
$\varepsilon_{ve}$: Elastic volume strain
$\varepsilon_{vc}$: Fracture volume strain
$\sigma_{ci}$: Crack closure stress
$$\sigma_{cd}$$: Damage stress
$$\sigma_{f}$$: Peak stress
$K_{I}$,${ }K_{{{\text{II}}}}$: Mode I and mode II stress intensity factors
a: Crack length
β: The angle between the stress and the original crack
$$\theta_{0}$$: Fracture angle
r,${\uptheta }$: Polar coordinate components
$\sigma_{i} \left( {i = r,{\uptheta }} \right)$: Crack tip stress components in polar coordinates
$\tau_{{r{\uptheta }}}$: Crack tip shear stress component in polar coordinates
$\sigma_{max}$: Maximum circumferential stress
D(α): Joint effect coefficient
$\sigma_{\alpha }$: The uniaxial compressive strength of the rock when the joint inclinations is α
$\sigma_{0}$: The uniaxial compressive strength of the rock when the joint inclinations is 0

## References

[1] Bieniawski ZT (1967). Mechanism of brittle fracture of rock, part I-theory of the fracture process. Int. J. Rock Mech. Mining Sci. Geomech. Abstracts.

[2] Taliercio A, Landriani GS (1988). A failure condition for layered rock. Int. J. Rock Mech. Mining Sci. Geomech..

[3] Ramamurthy T (1994). Engineering performance of phyllite foreign. Sci. Technol..

[4] Eberhardt E, Stimpson B, Stead D (1999). Effects of grain size on the initiation and propagation thresholds of stress-induced brittle fractures. Rock Mech. Rock Eng..

[5] Nicksiar M, Martin CD (2014). Factors affecting crack initiation in low porosity crystalline rocks. Rock Mech. Rock Eng..

[6] Tien YM, Kuo MCA (2001). Failure criterion for transversely isotropic rocks. Int. J. Rock Mech. Mining Sci..

[7] Zheng D, Ju NP (2011). Study on micro-fracture mechanism and fracture characteristics of Phyllite rock. J. Eng. Geol..

[8] Wang F, Meng LB, Li TB (2014). The influence of joints on physical and mechanical properties of thousands of rocks. Ind. Constr..

[9] Wu YS (2017). Research on the Mechanical Properties of Surrounding Rock of Phyllite Tunnel and Its Engineering Application.

[10] Wu YS, Tan ZS, Yu XB (2017). Comparative experimental study on strength and deformation characteristics of Phyllite in the northern section of Longmen Mountains. Chin. J. Geotechn. Eng..

[11] Wu YS, Tan ZS, Yu Y (2018). Anisotropic mechanical properties of a group of Phyllites in Maoxian County, Northwest Sichuan province. Rock Soil Mech..

[12] Wu YS, Zhongsheng T, Xianbin Y (2017). Expansion characteristics of uniaxial compression Phyllite under different loading azimuths. Rock Soil Mech..

[13] Zhou Y, Su SR, Li P (2019). Microstructure and mechanical properties of slab cracked Phyllite. J. Jilin Univ. (Earth Sci. Ed.).

[14] Martin CD, Chandler NA (1994). The progressive fracture of lac du bonnet granite. Int. J. Rock Mech. Mining Sci. Geomech. Abstracts.

[15] Zhang XP, Wang SJ, Han GY (2011). Experimental study on crack propagation under uniaxial compression of rock-A case study of flaky rocks. Chin. J. Rock Mech. Eng..

[16] Zhang XP (2010). Study on Deformation Failure Process and Strength Characteristics of Plate Structures Described by danba dimica Quartz Schist.

[17] Li Q, Hou J, Wang MY (2016). Experimental study on progressive failure mechanical properties of weakly cemented sandy mudstone. J. China Coal Soc..

[18] Behnia M, Goshtasbi K, Fatehi Marji M (2011). On the crack propagation modeling of hydraulic fracturing by a hybridized displacement discontinuity/boundary collocation method. J. Mining Environ..

[19] Behnia M, Shahraki AR, Moradian Z (2018). Equivalent strength for intact rocks in heterogeneous rock masses. Geotech. Geol. Eng..

[20] Aboutaleb S, Bagherpour R, Behnia M (2017). Combination of the physical and ultrasonic tests in estimating the uniaxial compressive strength and Young's modulus of intact limestone rocks. Geotech. Geol. Eng..

[21] Mokhtari M, Behnia M (2019). Comparison of LLNF, ANN, and COA-ANN techniques in modeling the uniaxial compressive strength and static Young's modulus of limestone of the dalan formation. Nat. Resour. Res..

[22] Alneasan M, Behnia M, Bagherpour R (2019). Analytical investigations of interface crack growth between two dissimilar rock layers under compression and tension. Eng. Geol..

[23] Mahmoud A, Mahmoud B, Raheb B (2020). Applicability of the classical fracture mechanics criteria to predict the crack propagation path in rock under compression. Eur. J. Environ. Civil Eng..

[24] Marji MF, Hosseini-Nasab H, Kohsary AH (2007). A new cubic element formulation of the displacement discontinuity method using three special crack tip elements for crack analysis. JP J. Solids Struct.

[25] Marji MF (2014). Numerical analysis of quasi-static crack branching in brittle solids by a modified displacement discontinuity method. Int. J. Solids Struct..

[26] Nikadat N, Marji MF (2016). Analysis of stress distribution around tunnels by hybridized FSM and DDM considering the influences of joints parameters. Geotech. Geol. Eng..

[27] Aliha MRM, Bahmani A (2017). Rock fracture toughness study under mixed mode I/III loading. Rock Mech. Rock Eng..

[28] Aliha MRM, Ayatollahi MR (2014). Rock fracture toughness study using cracked chevron notched Braziliandisc specimen under pure modes I and II loading—A statistical approach. Theoret. Appl. Fract. Mech..

[29] Aliha MRM, Mousavi SS, Ghoreishi SMN (2019). Fracture load prediction under mixed mode I plus II using a stress based method for brittle materials tested with the asymmetric four-point bend specimen. Theoret. Appl. Fract. Mech..

[30] Akbardoost J, Ayatollahi MR, Aliha MRM, Pavier MJ, Smith DJ (2014). Size-dependent fracture behavior of Guiting limestone under mixed mode loading. Int. J. Rock Mech. Min. Sci..

[31] Yang L, Peitian He, Mingji Z (2006). Research on rock failure mechanism based on damage fracture theory. J. Underground Space Eng..

